# Uropygial gland microbiota differ between free-living and captive songbirds

**DOI:** 10.1038/s41598-022-22425-4

**Published:** 2022-10-31

**Authors:** L. A. Grieves, C. L. J. Bottini, G. B. Gloor, E. A. MacDougall-Shackleton

**Affiliations:** 1 Department of Biology, The University of Western Ontario, 1151 Richmond St., London, ON N6A 5B7 Canada; 2 Present Address: Department of Biology, McMaster University, 1280 Main St. W, Hamilton, ON L8S 3L8 Canada; 3 Department of Biochemistry, The University of Western Ontario, 1151 Richmond St., London, ON N6A 5C1 Canada

**Keywords:** Ecology, Microbiology

## Abstract

Symbiotic microbes can affect host behavior and fitness. Gut microbiota have received the most study, with less attention to other important microbial communities like those of scent-producing glands such as mammalian anal glands and the avian uropygial gland. However, mounting evidence suggests that microbes inhabiting scent-producing glands play an important role in animal behavior by contributing to variation in chemical signals. Free-living and captive conditions typically differ in social environment, food diversity and availability, disease exposure, and other factors—all of which can translate into differences in gut microbiota. However, whether extrinsic factors such as captivity alter microbial communities in scent glands remains an open question. We compared the uropygial gland microbiota of free-living and captive song sparrows (*Melospiza melodia*) and tested for an effect of dietary manipulations on the gland microbiota of captive birds. As predicted, the uropygial gland microbiota was significantly different between free-living and captive birds. Surprisingly, microbial diversity was higher in captive than free-living birds, and we found no effect of dietary treatments on captive bird microbiota. Identifying the specific factors responsible for microbial differences among groups and determining whether changes in symbiotic microbiota alter behavior and fitness are important next steps in this field.

## Introduction

Symbiotic microbes can have diverse impacts on their hosts, including effects on development, endocrine state, immune function, nutrient availability, and behavior^1,2^. By recognizing the important role of microbiota in host fitness and capitalizing on technological advances that have made microbial DNA sequencing more accessible, our knowledge of wildlife microbial ecology is rapidly expanding^3^. Due to their importance in health, disease, and even behavior, the gut microbiota of both humans and wildlife has become increasingly well studied^4–7^. By contrast, we know far less about other glandular microbiota, despite mounting evidence that such regions also harbour diverse microbial communities that play an important role in animal behavior^8–11^.

The majority of wildlife microbial research in vertebrates has focused on mammals^9,12,13^. In these taxa, glandular microbiota can differ between the sexes and with age, group membership, and social and reproductive status^9,14–20^. For example, correlative studies suggest that anal gland microbiota are involved in the production or modification of olfactory signals of group membership in spotted and striped hyenas (*Crocuta crocuta*; *Hyaena hyaena*) and red foxes (*Vulpes vulpes*)^15,16,19^, social status in meerkats (*Suricata suricatta*)^14,21^, and reproductive status in spotted hyenas^16^. In birds, the main odor-producing gland is the uropygial gland, which similarly harbors diverse microbial communities^11,22^. While less well studied, the glandular microbiota of birds have similarly been shown to differ among sexes, age classes, populations, and with genotype in song sparrows (*Melospiza melodia*) and dark-eyed juncos (*Junco hyemalis*)^22–24^, and experimental evidence links uropygial gland microbiota to the production and/or modification of avian body odors in dark-eyed juncos and European hoopoes (*Upupa epops*)^25,26^. Furthermore, birds are capable of perceiving and responding to these odors^27–29^. Together, these findings suggest that symbiotic microbes may influence host social behavior through the production or modification of olfactory signals^9,11,25^.

Host immune genotype explains some of the variation in microbial community composition. For example, major histocompatibility complex (MHC) genotype predicts uropygial gland microbiota in song sparrows^24^ and male Leach’s storm petrels (*Oceanodroma leucorhoa*)^30^, and feather microbiota in blue petrels (*Halobaena caerulea*)^31^. However, extrinsic environmental factors also shape host microbiota. For example, uropygial gland microbiota differ in song sparrows from different breeding populations^22^ and feather microbiota differs between migratory and nonmigratory species^32^, suggesting that different habitat and climatic conditions affect microbial community composition.

To better understand the causes and consequences of variation in vertebrate host microbiota, within-species comparisons between free-living and captive populations are useful. Environmental and social conditions can differ markedly between natural and captive environments, providing an opportunity to better understand how such conditions affect host microbiota. For example, free-living and captive animals typically experience different diets and food availability, with free-living animals generally having access to greater food diversity with variable availability and captive animals receiving lower food diversity (e.g., nutritionally complete dietary preparations) ad libitum. Free-living animal species may experience temporal feast or famine conditions wherein diet type, diet richness, and food availability changes throughout the annual cycle, which can affect species’ microbiota. For example, in large herbivorous African mammals, dietary and microbial richness are not correlated, but dietary and microbial similarity are^33^. Free-living and captive animals typically also experience different levels of environmental complexity, different photoperiodic and temperature conditions, and different opportunities for social interaction with conspecifics. Captive animals also tend to experience more human interaction and handling than free-living animals and may also receive medications or other treatments that can affect their microbiota.

Most of what we know about how captivity alters the microbiota of wild animals comes from studies of the gut^13^. However, there is a paucity of data on how other glandular microbial communities—such as anal and uropygial gland microbiota, known to be important in animal communication and fitness—are affected by captivity. To address this gap, we used 16S rRNA gene sequencing to compare the composition and diversity of uropygial gland microbiota of free-living song sparrows versus those held in captivity under standardized conditions. For this study, we had two main predictions. First, based on the gut microbiome literature, we predicted significant differences in the uropygial gland microbiota of free-living and captive birds. Second, because song sparrows reside in diverse habitats and our study population is seasonally migratory^34^ (thereby increasing their likelihood of encountering and acquiring different microbes^32^) we predicted microbial diversity would be higher in free-living than captive birds.

The captive birds used in this study were wild-caught as part of another project exploring the effects of dietary methylmercury and unpredictable food stress on songbirds, so we took advantage of this experimental design to test for effects of these dietary treatments on the uropygial gland microbiota. Dietary exposure to heavy metals (e.g., mercury, lead, and cadmium) alters the gut microbiota of mice and rats^35–37^, and fat-soluble metals such as methylmercury can accumulate in fatty tissues like the uropygial gland (discussed in^38^), so we predicted that methylmercury exposure would alter uropygial gland microbiota. In humans, food insecurity (disrupted food intake or alterations in eating patterns) is associated with changes in the gut microbiota (e.g., higher fecal proportion of Proteobacteria and lower fecal proportion of Bacteroidetes in malnourished compared to healthy children)^39,40^, and could be considered analogous to the unpredictable food stress treatment we applied to song sparrows. Relatedly, experimentally elevating corticosterone (a proxy for stress) altered the gut microbiota of yellow-legged gulls (*Larus michahellis*; e.g., by decreasing the abundance of potentially beneficial phyla like Firmicutes as well as potentially pathogenic genera like *Mycoplasma* and *Microvirga*)^41^. Thus, stress is associated with changes to the gut microbiota of both mammals and birds. Because birds’ diet can affect the composition of gut microbiota^42–44^ and of uropygial gland secretions (preen oil)^45^, we expected unpredictable food stress to alter uropygial gland microbial community composition.

## Results

The 44 uropygial gland amplicon sequence variants (ASVs) we retained in free-living and captive song sparrows were distributed among 4 bacterial phyla, 7 classes, 13 orders, 26 families, and at least 27 genera (Table S1).

**Figure 1:**
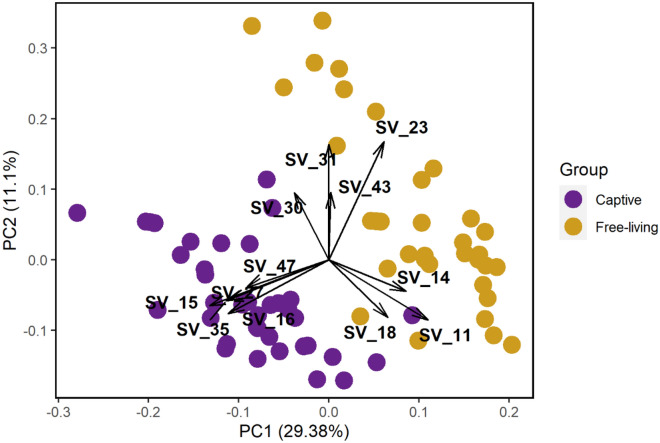
PC1 and PC2 scores derived from relative abundances of uropygial gland bacterial amplicon sequence variants (ASVs) sampled from free-living (n = 34) and captive (n = 37) song sparrows. Arrows indicate loadings based on ASV relative abundances that were most strongly associated with PC1 and PC2. Free-living birds had higher relative representation of ASVs 11, 14, 18, 23, 31, and 43 (Enterococcaceae, Clostridiales Family X1, Clostridiaceae 1, Comamonadaceae, Enterobacteriaceae, and Micrococcaceae respectively; upper right portion of graph). Captive birds had higher relative representation of ASVs 15, 16, 27, 35, and 47 (Gemmatimonadaceae, Pseudomonadaceae, Caulobacteraceae, Staphylococcaceae, and Methylobacteriaceae respectively; lower left portion of graph). Finally, two captive individuals had higher relative representation of ASV 30 (Comamonadaceae; midpoint of graph).

Bifactorial analysis of variance (ANOVA) tests indicated no significant differences in the uropygial gland microbial community composition of captive birds from different food treatment groups (Table 1). However, linear mixed effects models indicated significant differences in the uropygial gland microbiota of free-living and captive birds (Fig. 1, Table 2). Running linear mixed models using a fully paired design (analyzing only the subset of individuals for which we had both a free-living and captive sample) yielded similar results (Table S2).

**Table 1:** Results of ANOVA tests using factor scores from the first two principal components of PCA to test for differences in uropygial gland microbiota among treatment groups (dietary methylmercury, Y/N; unpredictable food stress, Y/N) in captive song sparrows (see Table S3 for factor loadings).

	df	Sum of squares	Mean sum of squares	F	*p*
**PC1**					
Mercury	1	15.8	15.8	0.5	0.470
Food stress	1	2.0	2.0	0.1	0.798
Residuals	34	1003.3	29.5	–	–
**PC2**					
Mercury	1	1.2	1.2	0.1	0.746
Food stress	1	0.9	0.9	0.1	0.782
Residuals	34	387.2	11.4	–	–

**Table 2:** Results of linear mixed effects models using factor scores from the first two principal components of PCA to test for differences in uropygial gland microbiota among free-living and captive song sparrows, using bird ID as a random effect to account for birds that were sampled twice: an initial (free-living) sample collected on the day of capture and a captive sample collected after approx. 11 months in captivity (see Table S3 for factor loadings).

	Estimate	SE	t	F	*p*
**PC1**					
Fixed effects					
Intercept	− 6.3	0.9	− 7.1	–	–
State (free-living, captive)	13.1	1.2	10.6	113.3	< 0.0001

	Variance	SD			
**PC1**					
Random effects					
Bird ID	2.8	1.7			
Residuals	25.9	5.1			

	Estimate	SE	t	F	p
**PC2**					
Fixed effects					
Intercept	2.4	0.7	3.3	–	–
State (free-living, captive)	− 5.1	1.1	− 4.7	22.1	< 0.0001

	Variance	SD			
**PC2**					
Random effects					
Bird ID	0.0	0.0			
Residuals	20.8	4.6			

Our principal component analysis (PCA) showed that the bacterial sequences most strongly associated with PC1 and PC2 were broadly associated with differences in the uropygial gland microbiota of free-living and captive birds (Fig. 1). Visual assessment of the PC1/PC2 biplot shows that free-living birds had higher relative representation of families Enterococcaceae, Clostridiales Family X1, Clostridiaceae 1, Comamonadaceae, Enterobacteriaceae, and Micrococcaceae (corresponding to more positive values of PC1 and PC2) and captive birds had higher relative representation of families Gemmatimonadaceae, Pseudomonadaceae, Caulobacteraceae, Burkholderiaceae, and Methylobacteriaceae (corresponding to more negative values of PC1). By contrast, we found no significant differences in uropygial gland microbiota based on dietary treatments (Table 1, Fig. S1). Component loadings of the first two principal components, retained for further analysis, are available in the supplementary material (Table S3). A permutational multivariate analysis of variance (PERMANOVA) test on the Euclidian distance matrix (compositional analysis) also identified significant differences in uropygial gland microbiota among captive and free-living birds but no effect of food treatment (Table 3), and a PERMANOVA test on the Bray–Curtis distance matrix (proportional analysis) yielded similar results (Table S4).

**Table 3:** Results of PERMANOVA using a Euclidean distance matrix to test for differences in uropygial gland microbiota among free-living and captive song sparrows and with dietary treatment while controlling for bird ID to account for birds that were sampled twice: an initial (free-living) sample collected on the day of capture and a captive sample collected after approx. 11 months in captivity.

Group	df	Sum of squares	Mean sum of squares	F	R^2^	*p*
State (free-living, captive)	1	3502.1	3502.1	18.2	0.21	< 0.0001
Mercury	1	209.6	209.6	1.1	0.01	0.170
Stress	1	148.2	148.2	0.77	0.01	0.742
Residuals	67	12 864.4	492.0	–	0.77	–

We detected 12 differentially abundant microbial taxa between captive and free-living birds. The microbial genus *Xylophilus* was elevated in free-living birds while the remaining 11 taxa were elevated in captive birds (Table 4). Of these differentially abundant taxa, five were the most highly distinguishable: the genera *Rhizobium*, *Caulobacter*, and *Bradyrhizobium*, and families Gemmatimonadaceae and Caulobacteraceae (Table 4; Fig. S2). There were no differentially abundant taxa among treatment groups in the captive birds.

**Table 4:** Differentially abundant microbial taxa (estimated effect size ≥ 1) among free-living and captive song sparrows calculated via a generalized linear model in ALDEx2. Benjamini–Hochberg adjusted p-values are reported. Italics indicate the most highly distinguishable taxa. A graphical representation is available in the supplementary materials (Fig. S2).

Family	Genus	Elevated in	Estimated effect size	Adjusted *p*
Comamonadaceae	Xylophilus	Free-living	1.04	0.0001
*Rhizobiaceae*	*Rhizobium*	*Captive*	*2.12*	*2.1* × *10*^*–18*^
*Gemmatimonadaceae*	*NA*	*Captive*	*1.94*	*3.9* × *10*^*–12*^
*Caulobacteraceae*	*Caulobacter*	*Captive*	*1.61*	*1.0* × *10*^*–9*^
*Caulobacteraceae*	*NA*	*Captive*	*1.58*	*1.2* × *10*^*–7*^
*Bradyrhizobiaceae*	*Bradyrhizobium*	*Captive*	*1.40*	*3.1* × *10*^*–5*^
Pseudomonadaceae	Pseudomonas	Captive	1.35	6.1 × 10^–8^
Sphingobacteriaceae	Mucilaginibacter	Captive	1.24	0.0003
Streptococcaceae	Lactococcus	Captive	1.17	0.0007
Oxalobacteraceae	Massilia	Captive	1.11	0.003
Methylobacteriaceae	Methylobacterium	Captive	1.10	0.0006
Burkholderiaceae	Ralstonia	Captive	1.01	0.0004

**Figure 2:**
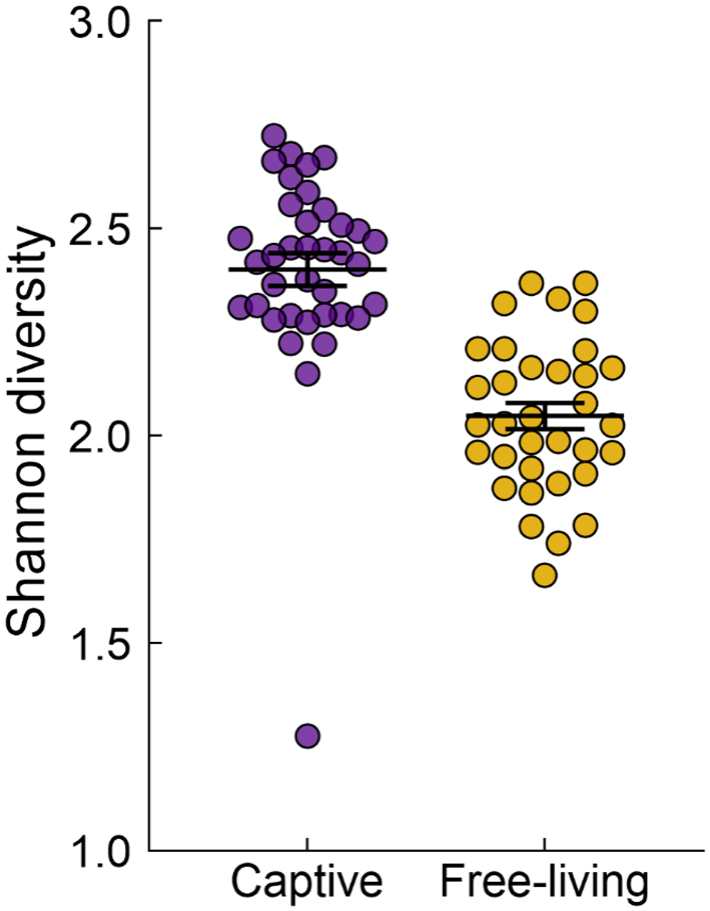
Shannon (alpha) diversity of uropygial gland microbiota was significantly higher in captive than free-living song sparrows. Bars denote mean ± SE.

The mean ± SE microbial Shannon (alpha) diversity of free-living and captive birds was 2.0 ± 0.03 and 2.4 ± 0.03 respectively, significantly higher in captive than free-living birds (F = 16.8, r^2^ = 0.40, p < 0.0001; Fig. 2). There was no difference in Shannon diversity among captive birds from different dietary treatment groups (F = 1.3, r^2^ = 0.11, p = 0.276; Fig. S3).

## Discussion

In support of our main prediction, uropygial gland microbial community composition was significantly different between captive and free-living song sparrows. Contrary to our secondary prediction, uropygial gland microbial diversity was higher in captive than free-living birds. While this diversity result was unexpected, some studies on the gut microbiota of non-avian vertebrates, and at least one study on avian gut microbiota, have similarly found greater microbial diversity among captive than free-living individuals^13,46^.

Overall, there are few studies on glandular microbial differences between free-living and captive birds, but several studies have found differences in the community composition of avian fecal microbiota, often used as a proxy for gut microbiota^42,43,46–48^. Fecal microbial diversity was higher in free-living than captive birds in capercaillies (*Tetrao urogallus*), rock ptarmigans (*Lagopus muta*), brown kiwis (*Apteryx mantelli*), and oriental white storks (*Ciconia boyciana*)^42,43,47,48^, while diversity was lower in free-living than captive individuals in red-crowned cranes (*Grus japonensis*)^46^. We are aware of only one other study that investigated changes in the uropygial gland microbiota of captive birds. In European hoopoes, uropygial gland microbial community composition, but not diversity, differed significantly between free-living and captive females, with free-living females having a significantly higher prevalence of bacteria from the family Veillonellaceae and the genus *Clostridium*^49^. Given the paucity of data on differences in the uropygial gland microbiota of free-living and captive birds, we focus our discussion on microbial differences in the gut microbiota of free-living and captive animals.

Cloacal microbiota are sometimes examined as a proxy for gut microbiota (e.g.^50,51^), but it should be noted that the cloaca receives inputs not only from the digestive system but also from the reproductive and urogenital systems. The avian cloaca may thus have a unique microbial community due to its role in receiving waste from multiple organ systems^52^ and may not accurately reflect the gut microbiota^53^. Nevertheless, cloacal microbiota differ between free-living and captive birds in some species^50,51^. Cloacal microbial diversity was higher in free-living than captive house sparrows (*Passer domesticus*)^51^ but lower in free-living than captive mealy parrots (*Amazona farinosa*), blue-and-yellow macaws (*Ara ararauna*), and red-and-green macaws (*Ara chloropterus*), though sample sizes for the parrot species studied were small^50^.

In non-avian vertebrates (fish, amphibians, reptiles, and mammals), most studies report differences in the gut microbial community composition and diversity of free-living and captive individuals, but there are no overarching directional patterns^13,54^. Instead, a recent meta-analysis that controlled for analytical differences by using a standardized bioinformatics pipeline demonstrated that differences in microbial composition and diversity seem to be primarily associated with site-specific conditions in captivity (e.g., diet, health treatments, environmental conditions, and level of contact with con- or heterospecifics), which differ across studies^13^. For example, captivity alters the gut microbiota in both white-throated and Stephen’s woodrats (*Neotoma albigula*; *N. stephensi*)^55^, but maintaining captive white-throated woodrats on a natural diet is associated with high (90%) retention of wild-type gut microbiota^56^. In contrast, woodrats fed an artificial diet retained only 62% of their wild-type gut microbiota^56^. Overall, captive populations tend to show an increase in human-associated microbiota compared to their free-living counterparts^13^. Whether or not such patterns hold for the gut and other glandular microbiota of birds warrants further study.

In contrast to our third prediction, dietary treatments of captive birds did not significantly alter their uropygial gland microbiota. We detected no effect of dietary methylmercury or unpredictable food stress on the uropygial gland microbiota of captive song sparrows. Dietary exposure (via drinking water) to the trace metal pollutants lead and zinc altered the plumage bacterial community composition of feral pigeons (*Columba livia*)^57^, so we speculated that dietary methylmercury might alter the uropygial gland microbiota, perhaps through accumulation of methylmercury in the gland or gland oil^38^. We previously detected an effect of unpredictable food stress, but not of dietary methylmercury, on the chemical composition of song sparrow preen oil^38^. Given that preen oil composition is affected by many factors, including uropygial gland microbiota^25,26,58^, we expected to see an effect of diet on gland microbiota. The chemicals that make up preen oil are thought to be secreted by uropygial cells after processing dietary substrates^59^, which may in turn influence the suitability of the gland environment for microbial colonization, thereby influencing uropygial gland microbial composition. While we cannot rule out diet (natural “wild-type” diet obtained by free-living birds vs the nutritionally complete agar diet administered in captivity) as a contributing factor to the uropygial gland community compositional and diversity differences we observed between free-living and captive song sparrows, the dietary treatments administered to captive birds in our experiment did not affect their uropygial gland microbiota.

In addition to dietary effects, other potential explanations for the differences we observed in the uropygial gland microbial community composition and diversity of free-living and captive song sparrows are human interactions and environmental conditions. Song sparrows were handled periodically (approx. once weekly) as part of animal care and other research procedures, fresh food and water was placed in cages daily, and cages and holding rooms were cleaned regularly. These types of interactions could have altered the microbial community composition and potentially also increased the microbial diversity of captive compared to free-living birds via horizontal transfer of microbes from humans to our study subjects^13^, though the likelihood that such microbes would reach and colonize the uropygial gland, a small organ located at the base of the tail and covered by body feathers, has not been evaluated. Moreover, the microbial taxa that were elevated in captive birds are not typical of human-associated microbes^60–62^.

Of the taxa that were elevated in captive birds, most (8/11) have previously been identified from the uropygial gland of free-living song sparrows and other avian species^22^. Three genera, *Mucilaginibacter*, *Lactococcus*, and *Massilia* have not previously been identified from song sparrows^22^, but *Mucilaginibacter* and *Lactococcus* have been found associated with dark-eyed juncos^23^. *Massilia* has mainly been identified from environmental sources, but also in animal feces^63^, while *Mucilaginibacter* is primarily found in soils and *Lactococcus* in plants^64^. Interestingly, some *Mucilaginibacter* species have been isolated from environments contaminated with heavy metals and are considered heavy metal resistant^65,66^. This raises the intriguing, albeit speculative, possibility that this genus was elevated in captive birds as a result of the dietary methylmercury treatment we applied to a subset of these birds, and then spread throughout the captive population through cross-contamination among cages within the holding facility. On a similar note, *Lactococcus* are lactic acid fermenting bacteria often found in dairy and other food products^67^ and may have been introduced through the agar-based diet, which contained the milk product casein.

The taxa that were most highly distinguishable (i.e., elevated) in captive birds (family Gemmatimonadaceae and genera *Rhizobium*, *Bradyrhizobium*, and *Caulobacter*) are also predominantly known from environmental sources. *Rhizobium* and *Bradyrhizobium* are typically found as nitrogen-fixing bacteria in soil, *Caulobacter* are known from freshwater lakes and rivers, and bacteria in the family Gemmatimonadaceae are widely distributed in nature^64,68^. While we cannot assign a source or cause directly, the observed differences in uropygial gland microbial community composition and diversity are most likely due to differences in diet and/or environmental conditions between the captive and free-living birds in our study, as has been found in studies of differences in the gut microbiota of free-living and captive animals^13,47,49^.

Symbiotic microbiota can influence animal health^69^ and behavior^70,71^. In this study, we detected significant differences in the uropygial gland microbial community composition and diversity of free-living and captive song sparrows. Uropygial gland microbiota may convey information about genetic compatibility and/or relatedness^24,30^, and have been implicated in the production and/or modification of avian odors that are involved in social and reproductive signaling^25^. It is therefore important to understand how different environmental conditions shape the symbiotic microbiota of birds, and the subsequent effects on behavior and fitness. Future studies on the glandular microbiota of free-living and captive birds should attempt to link changes in microbiota to changes in body odor, behavior, and reproduction.

## Methods

### Study subjects and housing

We used 49 adult song sparrows in this study. Of these, 39 (27 male, 12 female) were captured on their breeding territories in London, Ontario, Canada (42°59′5.64″ N, 81°14′43.08″ W) between 8 August–1 September 2017 and held in captivity overwinter. The remaining 10 (9 male, 1 female) were captured in London between 9–11 April 2018 and held in captivity as well. We captured birds via mist nets, using playback of adult song and juvenile distress calls to attract song sparrows to the nets. These birds were part of unrelated experiments^28,29,38,72^ and were used in this study in accordance with guidelines to reduce the number of animals used in research wherever possible (Canadian Council on Animal Care, CCAC).

We housed birds in individual cages at 20–22 °C with relative humidity of 30–70%. Birds were kept under a simulated natural photoperiod (approximately 13L:11D in April–May to 15L:9D in June–July) and with ad libitum access to water and food (Living World Premium Mix for Budgies parakeet seed mixed with ground Mazuri small bird diet) until 16 April 2018, when we began transitioning the birds to a nutritionally complete agar-based synthetic diet (containing 60% carbohydrate, 13.4% protein, and 10.6% lipid, dry mass basis; instructions and details in^38^). This diet was the birds’ major food as of 30 April 2018, except that a small quantity of blended eggs and bread (mean ± SE = 6.3 ± 0.1 g) or 2–4 mealworms was supplied once a week.

As part of other studies^38,72^, 37 of the 49 captive birds used in this study were assigned to four treatment groups beginning on 15 May 2018 and continuing until 10 July 2018: dietary methylmercury exposure (n = 13, hereafter ‘mercury’), unpredictable food stress (n = 9, hereafter ‘stress’), combined exposure to methylmercury and food stress (n = 9, hereafter ‘both’), and birds fed uncontaminated agar-based diet without unpredictable food stress (n = 6, hereafter ‘control’). Further details of these treatments can be found in^38^.

### Sample collection

To characterize the uropygial gland microbiota of free-living and captive song sparrows, we swabbed their uropygial glands for genetic analysis (details below) shortly after capture (hereafter ‘free-living sample’) and/or later in captivity (‘captive sample’). Of the 49 birds used in this study, 12 had a single free-living sample collected, 15 had a single captive sample collected, and 22 had both free-living and captive samples collected (Table S5). Free-living samples were collected between 8 August–1 September 2017 from birds within 6 h of being brought into captivity. Captive samples were collected on 9–10 July 2018, after 8 weeks of experimental dietary treatments (performed as part of a separate study^38,72^ from birds that were in captivity for approx. 11 months [27 birds captured in 2017] or 3 months [10 birds captured in 2018]). We thus used a within-study design where most free-living birds were sampled initially upon capture, then sampled again after being held in captivity for a minimum of 3 months. This staggered collection method allowed us to sample free-living and captive birds at approximately the same time of year across years (August 2017, July 2018), thus reducing the likelihood that sampling would be influenced by seasonal effects. All birds were sampled in the post-breeding period (determined by lack of brood patch and/or cloacal protuberance and based on knowledge of typical breeding and migration dates for this species^34,73^), thereby reducing the likelihood that sampling would be influenced by behavioral, hormonal, or other physiological effects associated with breeding. In migratory song sparrows, moult initiation dates are variable but usually occur between mid-June to mid-September^34^, so we do not think individual differences in moult status are likely to have affected our results.

Swabs were collected as follows. Each bird was handled using a fresh pair of nitrile gloves. First, we collected a preen oil sample from each individual for use in a separate study (i.e., not analyzed here) by gently probing the uropygial gland with a nonheparinized glass capillary tube^38^. Immediately after preen oil collection, we swabbed the uropygial gland by dipping a sterile medical grade swab into sterile molecular grade water then rubbing the swab firmly over and around the gland. This allowed us to collect microbes from both inside and outside the gland, assuming microbes from within the gland were excreted during preen oil sampling. We chose this approach because the small size of the uropygial gland in this species prevents noninvasively sampling microbes from directly or exclusively within the gland. However, since oil is frequently excreted from the gland, our external swabbing method was designed to collect bacteria inhabiting the gland as well as living immediately outside the gland, both of which are microbial sources that may be important for social and reproductive signaling in birds^24,74^. Swabbing was done using a continuous motion three times in each of the following directions: clockwise, counterclockwise, and up and down along the rostral/caudal axis. Swabs were stored in a sterile microfuge tube at − 20 °C pending analysis in March 2019 (i.e., after 8–19 months). We also collected a small blood sample (approx. 20 µL) from each bird through brachial venipuncture and used molecular analysis to sex all birds following^75^.

### Endpoints

This study was part of a separate experiment in which birds continued to be exposed to methylmercury and food stress treatments until 14 August 2018. Four birds died in captivity for reasons unrelated to the experiment. In accordance with our animal use protocols and CCAC guidelines to minimize pain or distress to animals^76^, all 45 remaining birds were euthanized via isoflurane inhalation (Fresenius Kabi) after completion of the larger study.

### DNA extraction and 16S amplification

We extracted bacterial DNA from swabs using DNeasy PowerSoil DNA isolation kits (Qiagen), consistent with previous work in this field^22–24^. Extractions were carried out in 14 batches of 24 (23 uropygial gland swabs plus one swab-only negative control per batch, including swabs used as part of separate studies; details in^22,24^). We amplified the V4 region of the bacterial 16S rRNA gene using the universal primers F518^77^ and R806^78^. Each primer included an Illumina MiSeq adaptor sequence, four randomized nucleotides, and a unique ‘barcode’ of eight nucleotides. We performed PCR in a total volume of 25 µL, including 10 µL of 5PRIME HotMasterMix (Quantabio), 0.2 µM of each primer, and 2 µL of DNA template ($\overline{{\text{x}}}$ concentration = 0.1 ng/µL, range = 0.01–0.12 ng/µL, measured using a Qubit fluorometer). The thermocycling profile was: 2 min at 94 °C; 35 cycles of 45 s at 94 °C, 60 s at 50 °C, and 90 s at 72 °C; and a 10 min final extension at 72 °C.

### Sequencing and pipeline

We pooled PCR products that showed amplification of the expected band size (approx. 300 nt) into a library and sequenced with 250 nt paired-end reads on an Illumina MiSeq at the London Regional Genomics Centre. We used a custom pipeline^79^ (workflow and parameters available at https://github.com/ggloor/miseq_bin/) and the R package dada2^80^ to process the raw reads. This involved the following steps: read demultiplexing, quality filtering and denoising, sequence variant inference, read overlapping, and removal of chimeras. Singleton ASVs were excluded by default in this pipeline. This produced a count table of ASVs by sample where ASVs rarer than 0.1% in any sample were removed following^81^. This resulted in an initial dataset of 1,826,620 reads containing 1690 ASVs from all 71 samples. We assigned each ASV to taxon by clustering at ≥ 97% sequence identity using the naïve Bayesian Ribosomal Database Project (RDP) Classifier^82^.

Of the 1690 ASVs in the initial dataset, most were very rare. Because rare sequences that occur in only a few samples are generally uninformative, and because samples with very low read counts are more likely to represent undersampling, we filtered sequences by the minimum proportion, minimum occurrence, and minimum sample count of reads. Sequences found in less than 0.5% of reads (consistent with MiSeq instrument error rates reported in^83^), fewer than 10% of samples, and samples with fewer than 5000 reads were removed following^22,24^, resulting in the retention of 87.5% of reads; for a total of 1,598,910 reads (i.e., the removal of 12.5% of reads) and the retention of 45 ASVs. For comparative purposes, we also tried a less stringent filtering of sequences found in less than 0.0005% of reads (consistent with methods reported for OTU and QIIME-based filtering approaches reported in^84^) and found no change in our results (Fig. S4). After removing one ASV identified as a chloroplast sequence, we obtained a final data set of 44 ASVs (Table S1) from 71 samples (34 free-living, 37 captive; mean ± SE retained reads per sample = 22,520 ± 1377).

High throughput sequencing generates relative abundance data, which have a constant, irrelevant sum. The number of reads is imposed by the capacity of the sequencing instrument, rendering these data compositional. Thus, the total number of reads obtained are not relevant to the interpretation of the data^85^. Instead, compositional data provide information about the relationship among components^86^. We therefore used a compositional data analysis approach that examines the read ratios between sequences^85,87,88^.

In most datasets observed and actual totals are not equal—this is due to missing components. Small values such as those below an instrument’s detection limit are often observed as zero. In such cases, zero counts reflect sampling or equipment limitations rather than true zeros. In reality, these counts are below a certain value, but the true value is unknown (i.e., these are left-censored data). Discarding or replacing these values with zero can lead to estimation bias, so values are typically imputed using an estimation method^89^. So, following^81^, we used Bayesian-multiplicative replacement to impute values for zero count sequences using the R package zCompositions^89^ then applied a centered log-ratio transformation to the zero-replaced data set, rendering the use of Euclidean distances meaningful in subsequent analyses^87,90^.

### Post-pipeline quality control

For initial data exploration we conducted a PCA of the centered log-ratio transformed data using zero-centered rotated variables and the ‘prcomp’ function in base R (following^87,91^). This allowed us to visually assess and identify any ASVs likely reflecting contamination by examining the resultant PC1 × PC2 biplot for ASVs associated specifically with contaminated controls (seven of 14 negative controls showed amplification of the expected product size). Finding none, we plotted all pairwise combinations of contaminated controls to further search for ASVs shared among controls, considering ASVs that fell on or near the 1:1 line of each biplot as likely contaminants. No ASVs were consistently common to contaminated control samples, so this approach identified no candidate contaminant sequences for removal.

As a complementary approach, we also tested for signatures associated with external contamination in the full (unfiltered) dataset using the frequency and prevalence methods in the R package decontam^92^. The frequency method identifies contaminants by comparing the frequency distribution of each ASV as a function of the input DNA concentration. In the contaminant model, the expected frequency varies inversely with total DNA concentration. In the non-contaminant model, the expected frequency is independent of the total DNA concentration^92^. Using the frequency method, we identified 51 of the initial 1690 ASVs as candidate contaminants, all of which were removed by our filtering steps. The prevalence method identifies contaminants by comparing the presence/absence of each ASV in samples to the presence/absence of each ASV in negative controls and is appropriate for low biomass samples such as ours^92^. We did not identify any candidate contaminants using this method (at three different thresholds: 0.5, 0.1, 0.05). Given the filtering and quality control steps we performed, we believe the reads observed in control samples were most likely due to cross contamination with the uropygial gland swabs (i.e., internal contamination^92^). Thus, we retained all ASVs that passed the aforementioned filtering and quality control steps.

### Data analysis

Statistical analyses were performed in R version 4.0.3^93^. We conducted a PCA of the centred log-ratio transformed data using zero-centered rotated variables and the ‘prcomp’ function in base R. Based on visual analysis of the PCA scree plot and the cumulative variance explained by the principal components, we retained the first two principal components which together accounted for 40.5% of the variance (Table S3). Visual assessment of qq-plots and residuals indicated that data and residuals were distributed approximately normally and the residuals showed no evidence of heteroscedasticity. Previously, we found no effect of sex on the uropygial gland microbiota of song sparrows, including the population used in this study^22^, so we pooled samples from both sexes for all analyses.

To test for heterogeneity of samples collected from captive birds that received different food treatments, we conducted two two-way ANOVA tests using presence/absence of methylmercury and presence/absence of food stress as the two predictor variables and factor scores from each of the two retained PCs as dependent variables. Based on these results, we used the package lme4^94^ to run two linear mixed effects models with PC1 and PC2 factor scores as dependent variables, and state (free-living vs captive, our main variable of interest) as a predictor variable. These mixed models included bird ID as a random factor because some individuals (22 of 49) were sampled twice, once at the time of capture and once after approx. 11 months in captivity. As a supplementary analysis, we re-ran the mixed effects model using only the subset of individuals that were sampled twice (i.e., a fully paired design). We also conducted a PERMANOVA on the Euclidean distance matrix using the ‘adonis’ command in the vegan package^95^ to test for an effect of state (captive, free-living) and diet treatment (presence/absence of methylmercury, presence/absence of food stress) on uropygial gland microbiota while controlling for bird ID. We visualized the distribution of microbial ASVs using a PCA biplot. Finally, we conducted a differential abundance test using a generalized linear model with the ALDEx2 (v1.6.0) package in Bioconductor^90,96,97^. We tested for differentially abundant taxa among free-living and captive birds as well as among treatment groups within the captive birds. We report taxa with an expected effect size difference ≥ 1 because effect size measures are more reproducible than P values^98^.

To compare our compositional method with proportional methods commonly used in the literature, we also analyzed the 16S rRNA gene sequencing data using another approach. We converted the raw read count data to proportions (rather than performing centered log-ratio transformations on the zero replaced data set), then conducted PERMANOVA on pairwise Bray–Curtis distance matrices constructed from the proportional 16S rRNA gene read count data to test for an effect of state (captive, free-living) and diet treatment (presence/absence of methylmercury, presence/absence of food stress) on uropygial gland microbiota while controlling for bird ID.

To evaluate microbial diversity among states and treatments we calculated Shannon (alpha) diversity using the ‘diversity’ function in the vegan package^95^. Then, using diversity as the response variable, we used linear models to test whether state and treatment predicted microbial diversity.

### Ethics approval

All applicable international, national, and/or institutional guidelines for the care and use of animals were followed. All birds were captured under permission from the Canadian Wildlife Service and Environment and Climate Change Canada (Scientific Collection Permit CA 0244; banding subpermits 10691E, F). All animal procedures were approved by The University of Western Ontario Animal Use Subcommittee (protocol # 2017-161). This study was conducted in accordance with ARRIVE guidelines (https://arriveguidelines.org/).

## Supplementary Information

Supplementary Information.

## Data Availability

16S rRNA gene sequencing files for free-living birds used in this study are available from the European Nucleotide Archive (ENA), study accession number: PRJEB42688 (sample_alias A17.##). Sequencing files for captive birds are available from the Sequence Read Archive (SRA), study accession number: PRJNA895414. Other supplementary data are available on Mendeley Data (10.17632/8d7vgk5pyy.3).
