# Supplementary material for: Uropygial gland microbiota differ between free-living and captive songbirds
Source: Sci Rep. 2022 Oct 31;12:18283. doi: 10.1038/s41598-022-22425-4 (PMC9622905; doi:10.1038/s41598-022-22425-4)
Supplement: Supplementary file 1 — Supplementary Information. [file 41598_2022_22425_MOESM1_ESM.pdf]

Supplementary Materials

Uropygial gland microbiota differ between free-living and captive songbirds

L.A. Grieves[1,2,*], C.L.J. Bottini[1], G.B. Gloor[3] & E.A. MacDougall-Shackleton[1]

[1]The University of Western Ontario, Department of Biology, 1151 Richmond St., London Ontario, N6A 5B7

[2]Current address: McMaster University, Department of Biology, 1280 Main St. W, Hamilton Ontario, L8S 3L8

[3]The University of Western Ontario, Department of Biochemistry, 1151 Richmond St., London, Ontario, N6A 5C1

[*]Corresponding author: grievel@mcmaster.ca, ORCID: 0000-0002-6836-2177

Supplementary Materials

Table S1. Taxonomic assignment of bacterial amplicon sequence variants (ASVs) collected from the uropygial gland of adult song sparrows.

SV	Phylum	Class	Order	Family	Genus
SV_41	Actinobacteria	Actinobacteria	Corynebacteriales	Corynebacteriaceae	Corynebacterium
SV_13	Actinobacteria	Actinobacteria	Corynebacteriales	Mycobacteriaceae	Mycobacterium
SV_28	Actinobacteria	Actinobacteria	Corynebacteriales	Nocardiaceae	Rhodococcus
SV_43	Actinobacteria	Actinobacteria	Micrococcales	Micrococcaceae	Micrococcus
SV_38	Firmicutes	Bacilli	Bacillales	Bacillaceae	Bacillus
SV_45	Firmicutes	Bacilli	Bacillales	Bacillaceae	Bacillus
SV_32	Firmicutes	Bacilli	Bacillales	Bacillaceae	—
SV_29	Firmicutes	Bacilli	Bacillales	Staphylococcaceae	Staphylococcus
SV_37	Firmicutes	Bacilli	Bacillales	Staphylococcaceae	Staphylococcus
SV_3	Firmicutes	Bacilli	Lactobacillales	Enterococcaceae	Enterococcus
SV_11	Firmicutes	Bacilli	Lactobacillales	Enterococcaceae	Enterococcus
SV_18	Firmicutes	Clostridia	Clostridiales	Clostridiaceae 1	Clostridium sensu stricto 13
SV_20	Firmicutes	Clostridia	Clostridiales	Clostridiaceae 1	Clostridium sensu stricto 3
SV_26	Firmicutes	Clostridia	Clostridiales	Eubacteriaceae	Eubacterium
SV_14	Firmicutes	Clostridia	Clostridiales	Family XI	Anaerosphaera
SV_24	Firmicutes	Clostridia	Clostridiales	Family XI	Anaerosphaera
SV_25	Firmicutes	Clostridia	Clostridiales	Lachnospiraceae	Lachnoclostridium
SV_36	Firmicutes	Clostridia	Clostridiales	Lachnospiraceae	Lachnoclostridium_5
SV_33	Firmicutes	Clostridia	Clostridiales	Ruminococcaceae	Oscillibacter
SV_15	Gemmatimonadetes	Gemmatimonadetes	Gemmatimonadales	Gemmatimonadaceae	—
SV_1	Proteobacteria	Alphaproteobacteria	Caulobacterales	Caulobacteraceae	Brevundimonas
SV_27	Proteobacteria	Alphaproteobacteria	Caulobacterales	Caulobacteraceae	Caulobacter
SV_35	Proteobacteria	Alphaproteobacteria	Caulobacterales	Caulobacteraceae	—
SV_12	Proteobacteria	Alphaproteobacteria	Rhizobiales	Bradyrhizobiaceae	Bradyrhizobium
SV_0	Proteobacteria	Alphaproteobacteria	Rhizobiales	Brucellaceae	Brucella
SV_6	Proteobacteria	Alphaproteobacteria	Rhizobiales	Methylobacteriaceae	Methylobacterium
SV_47	Proteobacteria	Alphaproteobacteria	Rhizobiales	Methylobacteriaceae	Methylobacterium
SV_7	Proteobacteria	Alphaproteobacteria	Rhizobiales	Rhizobiaceae	Rhizobium
SV_8	Proteobacteria	Alphaproteobacteria	Sphingomonadales	Sphingomonadaceae	Sphingomonas
SV_17	Proteobacteria	Alphaproteobacteria	Sphingomonadales	Sphingomonadaceae	Sphingomonas
SV_40	Proteobacteria	Betaproteobacteria	Burkholderiales	Burkholderiaceae	Ralstonia
SV_2	Proteobacteria	Betaproteobacteria	Burkholderiales	Alcaligenaceae	Castellaniella
SV_4	Proteobacteria	Betaproteobacteria	Burkholderiales	Alcaligenaceae	Castellaniella
SV_30	Proteobacteria	Betaproteobacteria	Burkholderiales	Comamonadaceae	—
SV_21	Proteobacteria	Betaproteobacteria	Burkholderiales	Comamonadaceae	Pelomonas
SV_23	Proteobacteria	Betaproteobacteria	Burkholderiales	Comamonadaceae	Xylophilus
SV_42	Proteobacteria	Gammaproteobacteria	Enterobacteriales	Enterobacteriaceae	—
SV_31	Proteobacteria	Gammaproteobacteria	Enterobacteriales	Enterobacteriaceae	Pantoea
SV_39	Proteobacteria	Gammaproteobacteria	Enterobacteriales	Enterobacteriaceae	Pantoea
SV_34	Proteobacteria	Gammaproteobacteria	Pseudomonadales	Moraxellaceae	Acinetobacter
SV_5	Proteobacteria	Gammaproteobacteria	Pseudomonadales	Pseudomonadaceae	Pseudomonas

SV_10	Proteobacteria	Gammaproteobacteria	Pseudomonadales	Pseudomonadaceae	Pseudomonas
SV_16	Proteobacteria	Gammaproteobacteria	Pseudomonadales	Pseudomonadaceae	Pseudomonas
SV_22	Proteobacteria	Gammaproteobacteria	Xanthomonadales	Xanthomonadaceae	Rhodanobacter

Table S2. Results of linear mixed effects models using factor scores from the first two principal components of PCA to test for differences in uropygial gland microbiota among free-living and captive song sparrows, using bird ID as a random effect to account for birds that were sampled twice: an initial free-living sample collected on the day of capture and a captive sample collected after approx. 11 months in captivity. Models were run only on the subset of 22 individuals for which we had a paired free-living and captive sample. See Table S3 for factor loadings.

	Estimate	SE	t	F	*p*
**PC1**					
Fixed effects					
Intercept	-6.6	1.1	-5.8	—	—
State (free-living, captive)	13.7	1.5	8.9	80.0	<0.0001
	Variance	SD			
Random effects					
Bird ID	2.8	1.7	—	—	—
Residuals	25.7	5.1	—	—	—
	Estimate	SE	t	F	*p*
**PC2**					
Fixed effects					
Intercept	2.1	1.0	2.1	—	—
State (free-living, captive)	-5.1	1.4	-3.6	12.8	0.0009
	Variance	SD			
Random effects					
Bird ID	0.0	0.0	—	—	—
Residuals	22.5	4.7	—	—	—

Supplementary Materials

Table S3 Eigenvalues, percentage of variance explained, and rotated component matrix for the first two principal components extracted from PCA analysis of song sparrow uropygial gland microbiota. Taxonomic assignment based on the Bayesian Ribosomal Database Project for each ASV is shown at the level of phylum and family. Bold text indicates ASVs strongly associated with each principal component. For complete taxonomic information see Table S1.

	PC1	PC2	Phylum	Family
Eigenvalue	71.6	26.5		
% variance explained	29.4	11.1		
SV_41	-0.058	0.098	Actinobacteria	Corynebacteriaceae
SV_13	0.067	-0.024	Actinobacteria	Mycobacteriaceae
SV_28	-0.121	0.028	Actinobacteria	Nocardiaceae
**SV_43**	**0.006**	**0.245**	**Actinobacteria**	**Micrococcaceae**
SV_38	0.073	0.006	Firmicutes	Bacillaceae
SV_45	-0.091	0.092	Firmicutes	Bacillaceae
SV_32	-0.041	0.144	Firmicutes	Bacillaceae
SV_29	-0.022	0.092	Firmicutes	Staphylococcaceae
SV_37	-0.193	0.012	Firmicutes	Staphylococcaceae
SV_3	0.135	-0.031	Firmicutes	Enterococcaceae
**SV_11**	**0.276**	**-0.209**	**Firmicutes**	**Enterococcaceae**
**SV_18**	**0.164**	**-0.201**	**Firmicutes**	**Clostridiaceae 1**
SV_20	0.146	-0.104	Firmicutes	Clostridiaceae 1
SV_26	0.142	-0.107	Firmicutes	Eubacteriaceae
**SV_14**	**0.215**	**-0.104**	**Firmicutes**	**Clostridiales, family XI**
SV_24	0.078	-0.072	Firmicutes	Clostridiales, family XI
SV_25	0.140	-0.148	Firmicutes	Lachnospiraceae
SV_36	0.116	0.013	Firmicutes	Lachnospiraceae
SV_33	0.114	-0.112	Firmicutes	Ruminococcaceae
**SV_15**	**-0.331**	**-0.160**	**Gemmatimonadetes**	**Gemmatimonadaceae**
SV_2	0.129	-0.029	Proteobacteria	Alcaligenaceae
SV_4	0.145	-0.074	Proteobacteria	Alcaligenaceae
SV_1	0.115	-0.022	Proteobacteria	Caulobacteraceae
**SV_27**	**-0.280**	**-0.139**	**Proteobacteria**	**Caulobacteraceae**
**SV_35**	**-0.284**	**-0.144**	**Proteobacteria**	**Caulobacteraceae**
SV_12	-0.152	-0.103	Proteobacteria	Bradyrhizobiaceae
SV_0	0.111	-0.012	Proteobacteria	Brucellaceae
SV_6	-0.027	0.190	Proteobacteria	Methylobacteriaceae
**SV_47**	**-0.230**	**-0.096**	**Proteobacteria**	**Methylobacteriaceae**
SV_7	-0.172	-0.005	Proteobacteria	Rhizobiaceae
SV_8	-0.070	0.160	Proteobacteria	Sphingomonadaceae
SV_17	-0.066	0.152	Proteobacteria	Sphingomonadaceae
SV_40	-0.199	-0.140	Proteobacteria	Burkholderiaceae
**SV_30**	**-0.095**	**0.253**	**Proteobacteria**	**Comamonadaceae**
SV_21	-0.041	-0.057	Proteobacteria	Comamonadaceae
**SV_23**	**0.153**	**0.429**	**Proteobacteria**	**Comamonadaceae**
SV_39	0.058	-0.030	Proteobacteria	Enterobacteriaceae

SV_42	-0.009	0.199	Proteobacteria	Enterobacteriaceae
**SV_31**	**0.000**	**0.416**	**Proteobacteria**	**Enterobacteriaceae**
SV_34	-0.070	0.072	Proteobacteria	Moraxellaceae
SV_5	0.129	-0.071	Proteobacteria	Pseudomonadaceae
SV_10	0.151	-0.036	Proteobacteria	Pseudomonadaceae
**SV_16**	**-0.280**	**-0.199**	**Proteobacteria**	**Pseudomonadaceae**
SV_22	0.110	-0.135	Proteobacteria	Xanthomonadaceae

Table S4 Results of PERMANOVA using a Bray-Curtis distance matrix to test for differences in uropygial gland microbiota among free-living and captive song sparrows and with dietary treatment while controlling for bird ID to account for birds that were sampled twice: an initial (free-living) sample collected on the day of capture and a captive sample collected after approx. 11 months in captivity.

Group	df	Sum of squares	Mean sum of squares	F	R^2	p
State (free-living, captive)	1	2.42	2.42	14.5	0.18	<0.0001
Mercury	1	0.09	0.09	0.55	0.01	0.957
Stress	1	0.10	0.10	0.60	0.01	0.769
Residuals	67	11.2	0.17	—	0.81	—

Supplementary Materials

Table S5. Summary data used in analysis of the uropygial gland microbiota of free-living and captive song sparrows. Free-living samples were collected at time of capture (August – September 2017). Captive samples were collected on 9 – 10 July 2018. Treatment refers to dietary treatments administered for 8 weeks prior to collection of the captive sample (mercury indicates dietary methylmercury, stress indicates unpredictable food stress, and M.S. refers to birds under both unpredictable food stress and dietary methylmercury; see main text for details).

**Total birds**	**Free-living only**	**Captive only**	**Free-living and captive**
49	12	15	22

**Sample label**	**Bird ID**	**Capture year**	**Sex**	**State**	**Treatment**	**Fate**
X1338.4	X1338	2018	M	Captive	Control	Euthanized (post experiment)
X1339.4	X1339	2018	F	Captive	Mercury	Euthanized (post experiment)
X1341.4	X1341	2018	M	Captive	Stress	Euthanized (post experiment)
X1346.4	X1346	2018	M	Captive	Stress	Euthanized (post experiment)
X1349.4	X1349	2018	M	Captive	M.S	Euthanized (post experiment)
X1350.4	X1350	2018	M	Captive	Mercury	Euthanized (post experiment)
X1353.4	X1353	2018	M	Captive	Stress	Euthanized (post experiment)
X56804.4	X56804	2018	M	Captive	M.S	Euthanized (post experiment)
X74951.4	X74951	2018	M	Captive	Stress	Euthanized (post experiment)
X74953.4	X74953	2018	M	Captive	Mercury	Euthanized (post experiment)
X75022.4	X75022	2017	M	Captive	M.S	Euthanized (post experiment)
A17.6	X75022	2017	M	Wild	Wild	Euthanized (post experiment)
X75024.4	X75024	2017	M	Captive	Control	Euthanized (post experiment)
A17.1	X75024	2017	M	Wild	Wild	Euthanized (post experiment)
X75025.4	X75025	2017	M	Captive	Stress	Euthanized (post experiment)
A17.2	X75025	2017	M	Wild	Wild	Euthanized (post experiment)
X75026.4	X75026	2017	M	Captive	Mercury	Euthanized (post experiment)
A17.3	X75026	2017	M	Wild	Wild	Euthanized (post experiment)
X75027.4	X75027	2017	M	Captive	M.S	Euthanized (post experiment)
A17.4	X75027	2017	M	Wild	Wild	Euthanized (post experiment)

Supplementary Materials

X75028.4	X75028	2017	F	Captive	Stress	Euthanized (post experiment)
A17.7	X75028	2017	F	Wild	Wild	Euthanized (post experiment)
X75029.4	X75029	2017	M	Captive	Mercury	Euthanized (post experiment)
A17.8	X75029	2017	M	Wild	Wild	Euthanized (post experiment)
A17.10	X75031	2017	M	Wild	Wild	Euthanized (post experiment)
X75032.4	X75032	2017	M	Captive	Mercury	Euthanized (post experiment)
A17.11	X75032	2017	M	Wild	Wild	Euthanized (post experiment)
A17.12	X75033	2017	M	Wild	Wild	Euthanized (post experiment)
A17.13	X75034	2017	M	Wild	Wild	Euthanized (post experiment)
X75035.4	X75035	2017	M	Captive	Stress	Euthanized (post experiment)
A17.14	X75035	2017	M	Wild	Wild	Euthanized (post experiment)
X75036.4	X75036	2017	M	Captive	Mercury	Euthanized (post experiment)
X75037.4	X75037	2017	M	Captive	Control	Euthanized (post experiment)
X75038.4	X75038	2017	M	Captive	Stress	Euthanized (post experiment)
A17.18	X75039	2017	F	Wild	Wild	Died (in captivity)
X75040.4	X75040	2017	F	Captive	Mercury	Euthanized (post experiment)
A17.19	X75040	2017	F	Wild	Wild	Euthanized (post experiment)
X75041.4	X75041	2017	M	Captive	Mercury	Euthanized (post experiment)
A17.20	X75041	2017	M	Wild	Wild	Euthanized (post experiment)
X75042.4	X75042	2017	M	Captive	M.S	Euthanized (post experiment)
A17.21	X75042	2017	M	Wild	Wild	Euthanized (post experiment)
X75043.4	X75043	2017	F	Captive	M.S	Euthanized (post experiment)
A17.22	X75043	2017	F	Wild	Wild	Euthanized (post experiment)
X74044.4	X75044	2017	F	Captive	Control	Euthanized (post experiment)
A17.23	X75044	2017	F	Wild	Wild	Euthanized (post experiment)
X75045.4	X75045	2017	M	Captive	Mercury	Euthanized (post experiment)
A17.24	X75045	2017	M	Wild	Wild	Euthanized (post experiment)
X75046.4	X75046	2017	M	Captive	M.S	Euthanized (post experiment)
A17.26	X75047	2017	F	Wild	Wild	Euthanized (post experiment)
A17.27	X75048	2017	M	Wild	Wild	Euthanized (post experiment)
X74049.4	X75049	2017	M	Captive	Control	Euthanized (post experiment)

Supplementary Materials

A17.28	X75049	2017	M	Wild	Wild	Euthanized (post experiment)
A17.29	X75050	2017	M	Wild	Wild	Euthanized (post experiment)
X75051.4	X75051	2017	M	Captive	Mercury	Euthanized (post experiment)
A17.30	X75051	2017	M	Wild	Wild	Euthanized (post experiment)
X75052.4	X75052	2017	F	Captive	Mercury	Euthanized (post experiment)
X75053.4	X75053	2017	M	Captive	M.S	Euthanized (post experiment)
A17.32	X75053	2017	M	Wild	Wild	Euthanized (post experiment)
A17.33	X75054	2017	F	Wild	Wild	Euthanized (post experiment)
X75055.4A	X75055	2017	F	Captive	Mercury	Euthanized (post experiment)
A17.34	X75055	2017	F	Wild	Wild	Euthanized (post experiment)
A17.35	X75056	2017	F	Wild	Wild	Died (in captivity)
A17.36	X75057	2017	F	Wild	Wild	Euthanized (post experiment)
A17.37	X75058	2017	M	Wild	Wild	Died (in captivity)
X75059.4	X75059	2017	M	Captive	Control	Euthanized (post experiment)
A17.38	X75059	2017	M	Wild	Wild	Euthanized (post experiment)
A17.39	X75060	2017	M	Wild	Wild	Died (in captivity)
X75061.4	X75061	2017	M	Captive	Stress	Euthanized (post experiment)
A17.40	X75061	2017	M	Wild	Wild	Euthanized (post experiment)
X75062.4	X75062	2017	F	Captive	M.S	Euthanized (post experiment)
A17.41	X75062	2017	F	Wild	Wild	Euthanized (post experiment)

Supplementary Materials

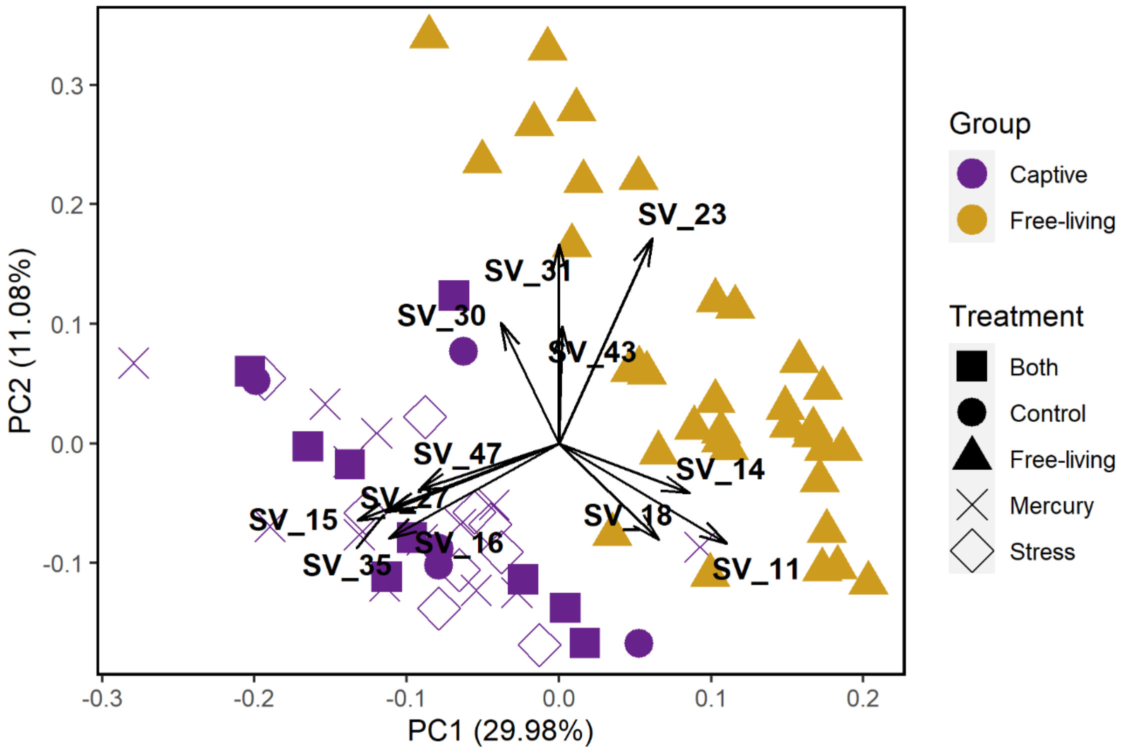

Fig. S1 PC1 and PC2 scores derived from relative abundances of uropygial gland bacterial ASVs sampled from captive (n = 37) and free-living (n = 34) song sparrows. As part of a separate study, the captive song sparrows were divided into four treatment groups: dietary methylmercury exposure (n = 13), unpredictable food stress (n = 9), both mercury and stress (n = 9), or an unmanipulated control group (n = 6). Treatment type had no effect on uropygial gland microbial composition (see main text for details).

Supplementary Materials

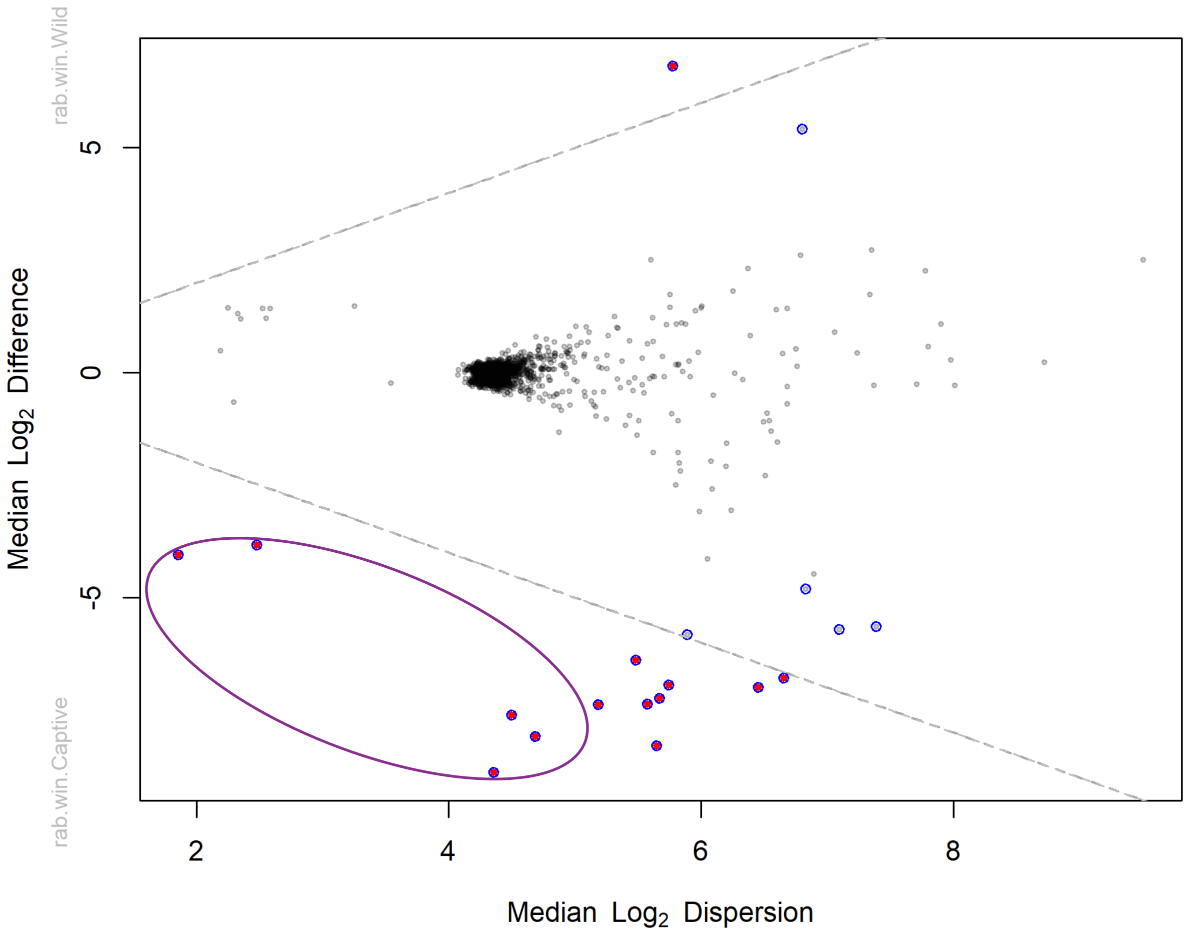

Fig. S2 Uropygial gland microbial taxa that were differentially abundant between captive and free-living song sparrows. Pink circles indicate taxa with an effect size ≥ 1 identified using a generalized linear mixed model while blue circles indicate additional taxa with an effect size ≥ 1 identified using t-tests. Taxa that were elevated in captive individuals have negative median $\log_2$ values (rab.win.Captive) while taxa elevated in wild individuals have positive values (rab.win.Wild). The five most highly distinguishable taxa are circled in pink. Black circles indicate taxa that were not differentially abundant between captive and free-living birds. See main text for details.

Supplementary Materials

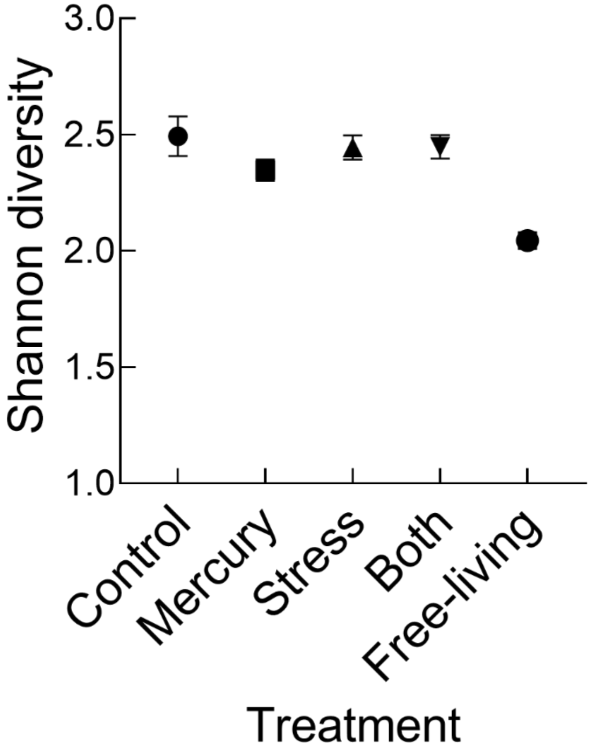

Fig. S3 Shannon (alpha) diversity of uropygial gland microbiota does not significantly differ among dietary treatment groups (control, methylmercury, unpredictable food stress, both mercury and stress) in captive song sparrows. By contrast, Shannon diversity significantly differs between free-living and captive birds (see main text for details). Values presented are mean ± SE.

Supplementary Materials

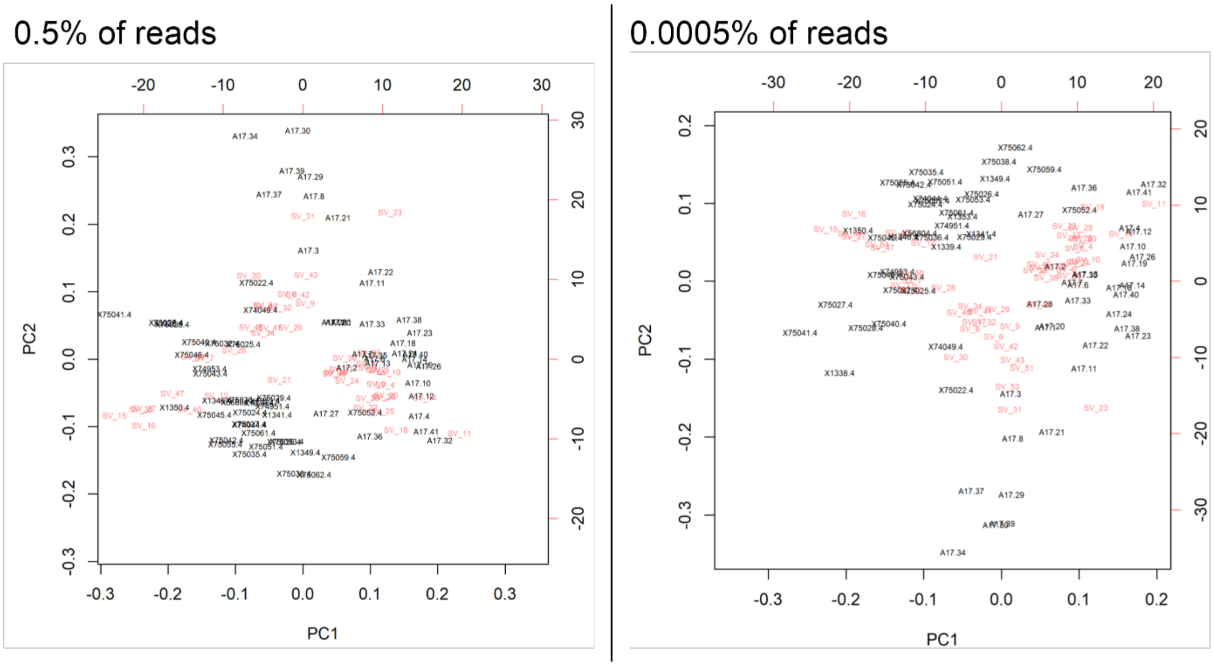

Fig. S4 Compositional biplot of samples (black font) and ASVs (red font) retained after removing sequences found in fewer than 10% of samples (i.e., minimum occurrence of reads), samples with fewer than 5000 reads (i.e., minimum sample count of reads), and sequences found in less than either 0.5% (i.e., minimum proportion of reads) or 0.0005% (i.e., a less stringent filtering approach) of reads. Note that rotations differ between plots but the overall position of samples and ASVs is robust to the choice of read-proportion threshold.
